# Effects of changes in end‐tidal PO_2_ and PCO_2_ on neural responses during rest and sustained attention

**DOI:** 10.14814/phy2.15106

**Published:** 2021-11-09

**Authors:** Tom Bullock, Barry Giesbrecht, Andrew E. Beaudin, Bradley G. Goodyear, Marc J. Poulin

**Affiliations:** ^1^ Department of Psychological and Brain Sciences University of California Santa Barbara California USA; ^2^ Institute for Collaborative Biotechnologies University of California Santa Barbara California USA; ^3^ Interdepartmental Graduate Program in Dynamical Neuroscience University of California Santa Barbara California USA; ^4^ Department of Physiology & Pharmacology University of Calgary Calgary Alberta Canada; ^5^ Hotchkiss Brain Institute Cumming School of Medicine University of Calgary Calgary Alberta Canada; ^6^ Department of Clinical Neurosciences University of Calgary Calgary Alberta Canada; ^7^ Department of Radiology University of Calgary Calgary Alberta Canada; ^8^ O’Brien Institute for Public Health University of Calgary Calgary Alberta Canada; ^9^ Libin Cardiovascular Institute of Alberta University of Calgary Calgary Alberta Canada; ^10^ Faculty of Kinesiology University of Calgary Calgary Alberta Canada

**Keywords:** arterial blood gasses, attention, EEG, neural oscillations, neurovascular coupling, P3 ERP

## Abstract

Impairments of cognitive function during alterations in arterial blood gases (e.g., high‐altitude hypoxia) may result from the disruption of neurovascular coupling; however, the link between changes in arterial blood gases, cognition, and cerebral blood flow (CBF) is poorly understood. To interrogate this link, we developed a multimodal empirical strategy capable of monitoring neural correlates of cognition and CBF simultaneously. Human participants performed a sustained attention task during hypoxia, hypercapnia, hypocapnia, and normoxia while electroencephalographic (EEG) activity and CBF (middle and posterior cerebral arteries; transcranial Doppler ultrasound) were simultaneously measured. The protocol alternated between rest and engaging in a visual target detection task that required participants to monitor a sequence of brief‐duration colored circles and detect infrequent, longer duration circles (targets). The target detection task was overlaid on a large, circular checkerboard that provided robust visual stimulation. Spectral decomposition and event‐related potential (ERP) analyses were applied to the EEG data to investigate spontaneous and task‐specific fluctuations in neural activity. There were three main sets of findings: (1) spontaneous alpha oscillatory activity was modulated as a function of arterial CO_2_ (hypocapnia and hypercapnia), (2) task‐related neurovascular coupling was disrupted by all arterial blood gas manipulations, and (3) changes in task‐related alpha and theta band activity and attenuation of the P3 ERP component amplitude were observed during hypocapnia. Since alpha and theta are linked with suppression of visual processing and executive control and P3 amplitude with task difficulty, these data suggest that transient arterial blood gas changes can modulate multiple stages of cognitive information processing.

## Graphical abstract

Changes in arterial blood gases (e.g., from high‐altitude exposure) are associated with impairments of a variety of cognitive functions, potentially via disruption of how blood flow responds to brain activity. We used a novel multimodal approach to simultaneously measure human brain electrical activity using electroencephalography and blood flow using transcranial Doppler ultrasound, while participants performed a demanding sustained attention task at varying levels of arterial oxygen and carbon dioxide (CO_2_). We observed changes in both spontaneous and task‐related brain activity as a function of changes in arterial CO_2_ as well as disruptions in neurovascular coupling, suggesting that transient changes in arterial CO_2_ can affect multiple stages of cognitive processing.

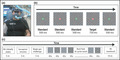

## 1 INTRODUCTION

Perturbations away from the normal arterial partial pressures of oxygen (PaO_2_) and carbon dioxide (PaCO_2_) can occur due to a wide range of chronic clinical conditions as well as transient environmental factors such as exposure to high altitude or physical exertion. Hypercapnia (elevated PaCO_2_) is prevalent in patients suffering from chronic obstructive pulmonary disease (COPD) and may also be observed in patients with obstructive sleep apnea (OSA; Jeffrey et al., 1992; Kaw et al., 2009). Moreover, hypocapnia (decreased PaCO_2_) is a known symptom of various pulmonary diseases, cardiovascular disorders, anxiety, pain, pregnancy, and exposure to high altitudes (Laffey & Kavanagh, 2002). Hypoxia is also associated with clinical disorders such as OSA and high‐altitude environments (Neubauer, 2001).

The relationship between cerebral blood flow (CBF) and neural activity is known as neurovascular coupling (Girouard & Iadecola, 2006), and there is evidence to show that changes in brain function that result from perturbations in CBF can have severe physiologic and psychologic consequences. For example, COPD is associated with cognitive dysfunction (Perry & Hodges, 1999; Schou et al., 2012), whereas OSA is associated with fatigue, fluctuations in mood, and deficits in attention and memory (Champod et al., 2013; Stierer & Punjabi, 2005). Exposure to hypoxic environments also impairs cognitive function, increasing the risk of accidents for individuals who engage in work or leisure at high altitude (De Aquino Lemos et al., 2012; Kramer et al., 1993; Virues‐Ortega et al., 2004). Personal and economic costs associated with these negative functional outcomes underscore the importance of determining the underlying physiologic mechanisms through which alterations in arterial blood gases impact brain function.

Understanding the link between cerebrovascular function and cognition requires approaches that can concurrently assess the neural and vascular components of neurovascular coupling as well as behavior. Several investigations have indirectly assessed this link by exploring the relationship between CBF and various facets of cognitive performance. For example, the enhanced vascular function has been linked to superior spatial reasoning, memory, processing speed, and inhibitory control function (Brown et al., 2010; Dupuy et al., 2015; Gauthier et al., 2015). Other studies have employed techniques from cognitive neuroscience to image the brain while volunteers perform cognitive tasks at different levels of PaO_2_ and PaCO_2_. For example, several investigations have used scalp electroencephalography (EEG) to monitor brain electrical activity while participants performed variants of an “oddball” task, during which they monitor a stream of visual or auditory stimuli for infrequent oddball targets (e.g., Polich, 2007; Polich & Criado, 2006). Both the latency of neural activity associated with target classification and detection and the behavioral response time to targets have been shown to increase as a function of hypoxia, suggesting impairment of stimulus processing (Fowler & Kelso, 1992; Fowler & Prlic, 1995; Hayashi et al., 2005; Singh et al., 2003; Thakur et al., 2011). Even when participants are not engaged in a task, changes in PaCO_2_ are associated with modulations in cortical blood oxygen level‐dependent functional magnetic resonance imaging (fMRI) measures (Wise et al., 2004). These investigations provide important insight, but without concurrent measurements of electrical activity and CBF, similar to those performed in nonhuman primates (Schmid et al., 2006; Zaidi et al., 2015), it is difficult to draw conclusions about changes in neurovascular coupling that underlie cognitive performance.

The objectives of the present study were thus to (1) assess neurovascular coupling in humans by combining EEG and transcranial Doppler ultrasound (TCD) measures of neurophysiology and CBF, respectively, at rest and during a cognitive task and (2) investigate the impact of changes in arterial blood gasses (i.e., hypoxia, hypercapnia, and hypocapnia) on neurovascular coupling in healthy participants performing a relatively simple, but attention‐demanding task. Attention supports many cognitive functions, and the ability to sustain attention to task‐relevant information and ignore distraction plays a critical role in many everyday activities (Corbetta et al., 2008; Lavie, 2005). Moreover, disturbances in attentional function are some of the first symptoms to surface in a variety of neurologic disorders (Parasuraman & Haxby, 1993; Perry & Hodges, 1999) and cognitive detriments following stroke (Mesulam, 1990). Thus, understanding the relationship between alterations in neurovascular coupling and attention will translate well to cognition in general.

## 2 METHODS

### 2.1 Ethical approval

This study was carried out in accordance with the *Declaration of Helsinki* and was approved by the Conjoint Health Research Ethics Board at the University of Calgary (REB14‐2287 and REB15‐0618). Prior to the study, all participants were informed of the study protocol and instrumentation and provided written informed consent.

### 2.2 Participants

Twelve healthy, normotensive participants (nine males, three females) volunteered for this study. The novel, multimodal nature of our approach meant that it was difficult to identify effect sizes from previous publications that we could use as the basis for formal power analysis. Accordingly, we based our sample size on prior work testing the effects of arterial blood gas manipulations on CBF using TCD measures (Ainslie & Poulin, 2004, *n* = 9; Poulin & Robbins, 1996, *n* = 6; Poulin et al., 1996, *n* = 6) as well as investigations that combine arterial blood gas manipulations with EEG measures (Kennealy et al., 1986, *n* = 10), MEG measures (Hall et al., 2011, *n* = 6), and cognitive tasks (Champod et al., 2013, Study 1: *n* = 8, Study 2: *n* = 9). Female participants were all tested during the follicular phase of their menstrual cycle. Participant characteristics are reported in Table 1. Participants had been living in Calgary, Alberta, Canada (~1045 m above sea level) for at least 1 year and were instructed to abstain from caffeine and physical activity for at least 4 h before the experimental session.

**TABLE 1:** Demographic data for all participants and information regarding exclusion from specific analyses

Participant	Sex	Age (years)	Handed	Height (cm)	Weight (kg)	BMI (kg/m^2^)	SBP (mm Hg)	DBP (mm Hg)	MBP (mm Hg)	Excluded from analysis	Reason
1	M	55	R	170	77.1	26.7	143	89	107		
2	M	36	R	180	92.5	28.5	114	78	90		
3	M	22	L	171	71.8	24.6	108	72	84	Control	Did not complete
										SSVEP	Incorrect monitor refresh rate
4	M	22	R	175	79	25.8	118	68	84		
5	M	33	R	181	85.6	26.1	110	75	87	Control	Did not complete
6	M	21	R	180	84	25.9	125	74	91	Behavior	Button presses not logged
7	M	28	R	175	70	22.9	110	63	79		
8	M	30	R	180	70.3	21.7	109	71	83		
9	M	42	R	169	65	22.9	115	77	90		
10	F	22	R	158	49.9	20.0	113	74	87		
11	F	32	R	157	58.1	23.6	104	68	80		
12	F	26	R	171	63	21.5	107	69	82	CBF, NVC	No PCA Hpo measure
	M = 9	31 (10)	11	172 (8)	72 (12)	24 (3)	115 (11)	73 (7)	87 (7)		

Abbreviations: BMI, body mass index; CBF, cerebral blood flow; DBP, diastolic arterial pressure; MAP, mean arterial pressure; NVC, neurovascular coupling; PCA, posterior cerebral artery; SBP, systolic arterial pressure; SSVEP, steady‐state visual evoked potential.

### 2.3 EEG data acquisition

EEG was measured using 19 gold disk scalp electrodes (Grass Technologies) according to the international 10–20 configuration. Electrode locations were measured and marked on the scalp with a wax pencil. The scalp was prepared for electrode placement by gentle abrasion with sandpaper and rearranging hair. The electrodes were attached using electrode paste (Ten20 Conductive Neurodiagnostic Electrode Paste, Weave and Company) and secured with gauze and collodion glue (Collodion Flexible, JT Baker). Electrode placements included: F3, Fz, F4, C3, Cz, C4, P3, Pz, P4, PO3, POZ, PO4, O1, OZ, O2, M1, and M2. An additional two electrodes were situated 10% anterior and 10% posterior to Cz to act as a reference and ground, respectively. Eye movements were monitored with horizontal and vertical electrooculogram electrodes (HEOG and VEOG, respectively). The HEOG electrodes were placed laterally from the left and right eyes, whereas the VEOG electrodes were placed on the skin superior and inferior to the left pupil. All electrode leads were plugged into a headbox (Compumedics Neuroscan SynAmps_RT_ Amplifier Headbox) connected to a personal computer that collected the EEG and EOG signals using MagLinkRT Neuroscan software.

### 2.4 Vascular measurements

Following application of the EEG electrodes, participants were instrumented with bilateral 2 MHz pulsed TCD probes held in place with snug‐fitting headgear (TOC2M, Multigon Industries Inc.). Blood velocity associated with the maximal frequency of the Doppler shift (i.e., peak velocity) through the middle cerebral artery (MCAv) was recorded from the contralateral hemisphere to the participant's dominant hand, and peak posterior cerebral artery blood velocity (PCAv; P2 segment) was assessed on the ipsilateral hemisphere. Finger blood pressure was measured continuously on the nondominant hand (Finometer Pro, Finapres Medical Systems, Amsterdam, Netherlands) and calibrated to the average systolic (SBP), diastolic (DBP), and mean (MAP) arterial brachial blood pressures measured intermittently on the contralateral arm (BP755; Omron Healthcare), prior to start of each behavioral task (Section 3.7). In addition, heart rate (3‐lead electrocardiogram (ECG); Micromon 7142B, Kontron Medical) and arterial oxyhemoglobin saturation (finger oximetry; 3900p, Datex‐Ohmeda) were measured continuously throughout all tests.

### 2.5 Gas instrumentation

**FIGURE 1:**
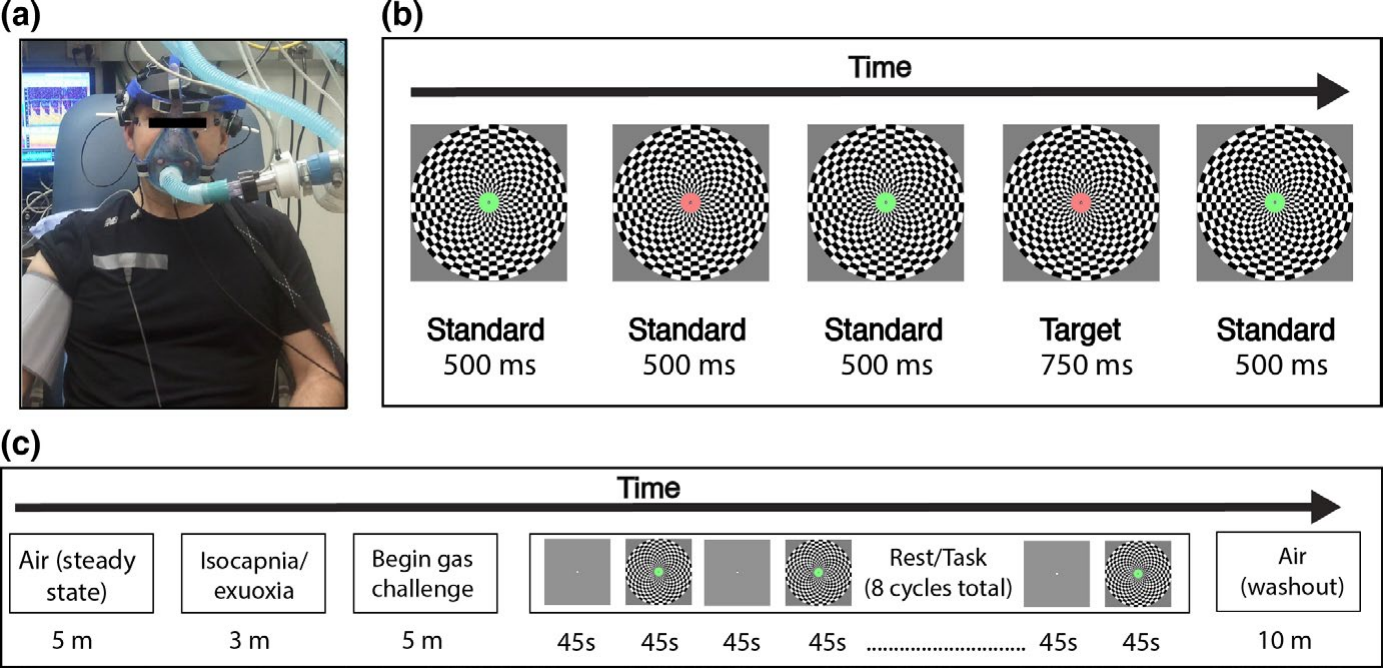
Methods (a) participant instrumentation. Participants were fitted with EEG electrodes, transcranial Doppler ultrasound, ECG, and a respiratory mask connected to a dynamic end‐tidal forcing system. (b) Schematic example of the behavioral task. During the “task” phase, participants monitored a centrally presented circular annulus that alternated between red and green. A button‐press response was made when a slightly longer duration target (10% of trials) was detected. A large checkerboard pattern was presented in the background with a contrast pattern that reversed every 8.33 Hz in order to drive a steady‐state response in the cortex. (c) Gas challenge protocol. Each gas challenge condition began with 5 min of air breathing to establish steady‐state breathing, followed by a 3‐min isocapnia‐euoxia baseline period, and then 5 min at the desired gas concentration to achieve steady‐state conditions before beginning the attention rest/task protocol. Participants then alternated between 45 s of rest (fixating on a white dot presented at the center of the screen) and 45 s of the behavioral task. After completing eight full cycles (12 min total), participants then breathed room air for 10 min (washout period)

Participants were instrumented with a full‐face, nonvented respiratory mask (Mirage NV Full Face Mask Series 2, Resmed) connected to a dynamic end‐tidal forcing (DEF) system. Using dedicated software (BreatheM v2.4; University Laboratory of Physiology, University of Oxford), the DEF system uses a negative feedback loop to control participants’ end‐tidal partial pressures of carbon dioxide (Pet_co_ _2_) and oxygen (Pet_o_ _2_) at desired levels independent of breathing rate and depth (Ainslie & Poulin, 2004; Vantanajal et al., 2007). See Figure 1a for an example of a fully instrumented participant.

### 2.6 Gas challenge conditions and protocol

The experimental session started with 10 min of air breathing to ascertain resting Pet_co_ _2_ and Pet_o_ _2_ values. Next, the participant performed visual stimulation challenges under four gas conditions with the order randomized and counterbalanced between participants. The four challenges were isocapnic‐euoxia (Pet_co_ _2_ = 1 mm Hg above resting values; Pet_o_ _2_ = 88 mm Hg [euoxic Pet_o_ _2_ for the altitude at which the laboratory was located]), euoxic‐hypercapnia (Pet_co_ _2_ = 8 mm Hg above resting values; Pet_o_ _2_ = 88 mm Hg), euoxic‐hypocapnia (Pet_co_ _2_ = 12 mm Hg below resting values; Pet_o_ _2_ = 88 mm Hg; performed using controlled [coached] hyperventilation), and isocapnic‐hypoxia (Pet_co_ _2_ = 1 mm Hg above resting values; Pet_o_ _2_ = 50 mm Hg). Compared with normoxia (i.e., air breathing), hypercapnia and hypoxia will increase CBF (Poulin & Robbins, 1996; Poulin et al., 1996; Xu et al., 2012), whereas hypocapnia will decrease CBF (Ito et al., 2003; Poulin et al., 1998). These gas challenges were used to interrogate the independent effects of changes in arterial pco_2_ and po_2_ on the interaction between neural activity, CBF, and performance on an attention task without the potential competing effect of simultaneous fluctuations in other blood gases resulting from gas challenge‐induced increases in ventilation (e.g., hyperoxia during hypercapnia and hyperventilation‐induced hypocapnia, and hypocapnia during hypoxia). In 10 participants, a fifth gas condition was included consisting of isocapnic‐euoxia with hyperventilation (Pet_co_ _2_ = 1 mm Hg above resting values; Pet_o_ _2_ = 88 mm Hg). This condition served as a control for the euoxic‐hypocapnia condition where hyperventilation was used to decrease Pet_co_ _2_. For the sake of brevity, the gas challenges are referred to in the remaining text and figures as isocapnic‐euoxia (IsoEu), hypercapnia (Hcap), hypocapnia (Hpo), hypoxia (Hpox), and hyperventilation (Hv) challenges.

Each gas challenge started with 5 min of air breathing to allow the participant to establish steady‐state breathing before starting the challenge protocol. Next, the participant underwent a 3‐min isocapnic‐euoxia baseline period, where Pet_co_ _2_ was maintained at 1 mm Hg above resting levels and Pet_o_ _2_ was held at 88 mm Hg. At the end of the baseline period, inspired concentrations of CO_2_ and O_2_ were adjusted to induce the desired changes in Pet_o_ _2_ and Pet_co_ _2_. The participant was held at each gas concentration for at least 5 min to achieve steady‐state conditions before starting the attention task protocol.

### 2.7 Behavioral task protocol

The participant was positioned 125 cm from a 19‐in. monitor. During each gas challenge, participants alternated between a *rest* phase and a *task* phase. The rest phase required the participant to gaze at a small white circle subtending 0.1° of visual angle presented at the center of a plain gray screen for 45 s. The rest phase was followed immediately by the task phase, where the participants were required to attend to a circular annulus (subtending 0.75° of visual angle) that continuously alternated between the colors red and green (Figure 1b). Each presentation of the green or red annulus constituted a “trial.” For 90% of trials, the duration of the red or green annulus was 500 ms (“standard”). For the remaining 10% of trials, the duration was 750 ms (“target”), and these trials were randomly distributed among the other 90%. Participants were instructed to press a mouse button as quickly and as accurately as possible when they detected a target. There were ~6 targets per 45 s task phase. During the task phase, the central annulus was superimposed over a large circular black and white checkerboard pattern. The checkerboard subtended 11.9° of visual angle and the contrast pattern was reversed at 8.33 Hz to generate a steady‐state visual evoked potential (SSVEP) that could be measured at the scalp using EEG (Norcia et al., 2015).

This rest‐task cycle was repeated eight times in total within the gas manipulation protocol (Figure 1c), resulting in ~48 targets per gas condition. Upon completion of the final cycle, the participant was returned to room air for at least 10 min as a washout phase prior to the start of the next gas challenge. Following instrumentation (~1.5 h), the study took ~3.5 h to complete. Note that prior to instrumentation, participants completed one full rest‐task cycle in order to familiarize themselves with the protocol and the target detection task.

The task was specifically designed to investigate the effects of different levels of arterial blood gases on multiple stages of neural and behavioral information processing. The detection task provided a behavioral response and allowed for the assessment of neural activity associated with later stages of cognitive processing pertaining to stimulus evaluation and classification (Polich, 2007; Polich & Criado, 2006), while the large, high contrast, inverting stimulus display drove activation in cortical regions associated with the early stages of visual processing (Di Russo et al., 2007) as well as driving a robust increase in CBF to the visual cortex (e.g., Feng et al., 2004; Uludag et al., 2004). The design meant that the effects of arterial blood gases on the tonic neural and vascular responses associated with the resting and task phases could be assessed as well as the event‐related neural responses associated with task performance.

### 2.8 EEG data analysis

#### 2.8.1 Preprocessing

MATLAB (version 2019a, Massachusetts, The MathWorks Inc.) was used for offline processing of the EEG data, along with the EEGLAB (version 14.1.1, Delorme & Makeig, 2004) and ERPLAB (version 7.0, Lopez‐Calderon & Luck, 2014) toolboxes. The continuous data were high‐ and low‐pass filtered at 0.1 and 30 Hz, respectively (slope 6 dB octave^−1^), and then ocular artifacts were removed using the Automatic Artefact Removal Toolbox (Gomez‐Herrero et al., 2006), available as an extension for EEGLAB.

#### 2.8.2 Continuous oscillations

Two different signal processing techniques were applied to investigate whether the different gas conditions modulated activity in the alpha frequency band and at the visual stimulation frequency (8.33 Hz). Alpha is typically measured between 8 and 12 Hz or 8–14 Hz, but in the present study, it was measured between 9 and 12 Hz to avoid capturing brain activity evoked at the stimulation frequency.

The first technique estimated alpha power at parieto‐occipital and occipital scalp regions over the duration of a complete 90 s rest/task cycle. Data from electrodes POz, PO3, PO4, Oz, O1, and O2 were parsed into 90 s epochs that spanned the complete rest/task cycle and then filtered using a third‐order Butterworth bandpass filter between 9 and 12 Hz to isolate the alpha band. A Hilbert Transformation was then applied to the data, allowing an estimate of instantaneous amplitude at each time point and scalp electrode. These instantaneous amplitude values were then converted to power (μV^2^) by taking the absolute value and squaring, and these values were then averaged across epochs.

The second technique estimated mean spectral power across a range of frequency bands during the rest and task phases, by transforming data from the time domain to the frequency domain. The data were parsed into epochs spanning the final 15 s of the rest and task phases, and the mean of the epoch was used as a baseline to be subtracted from each data point. A fast Fourier transform (FFT) was computed for each individual epoch and channel, and the complex output of the FFT was converted to power (μV^2^) by squaring the absolute value of the output. These values were then averaged across epochs. Alpha power was computed for the final 15 s of the rest and task phases by averaging data from 9 to 12 Hz. To control for any global shifts in spectral power across gas conditions (i.e., shifts in frequency bands outside alpha), a global baseline measure was computed by averaging theta and beta power and subtracting this from alpha power.

To assess the magnitude of the steady‐state response to the flickering checkerboard, power at the second harmonic of the stimulation frequency (16.67 Hz) was isolated and used in subsequent analyses. It has been established that the second harmonic of a constant pattern‐reversing stimulus has greater magnitude than the response at the stimulation frequency and peaks over the parieto‐occipital region of the scalp (Garcia et al., 2013; Norcia et al., 2015; Regan, 1989). To control for any global additive shifts in power as a function of gas challenge, a baseline‐corrected measure of steady‐state power was calculated by subtracting the mean of the surrounding 1 Hz frequency bins from the SSVEP peak.

Mean power computed across all parieto‐occipital and occipital electrodes (POz, PO3, PO4, Oz, O1, and O2) were used for both alpha and steady‐state analyses. Both baseline‐corrected and uncorrected measures were submitted to statistical tests. Due to a technical issue, the checkerboard oscillated at a different frequency for one participant during the Hpo condition. Thus, the data from this participant were not included in the steady‐state analysis.

#### 2.8.3 Event‐related activity

Two different signal processing techniques were used to test whether the different gas conditions modulated event‐related neural activity during the task phase of each trial.

First, to investigate the activity specifically related to target processing, EEG data from target trials were epoched from −100 to 700 ms around the target onset, which was defined as the time at which the target became distinct from the standard (i.e., 500 to the 750 ms target trial). A target trial was only included in this analysis if a response was made between 200 and 1500 ms post target onset. These trials were then submitted to threshold rejection, and trials exceeding ±75 μV across all scalp channels were excluded. Mean trials rejection rates were <10% across all conditions. Trials were then averaged to form an event‐related potential (ERP) for each subject and condition. The task was designed such that participants responded to rare targets among frequent standards (nontargets), as this task structure is known to elicit a robust P3 ERP component, typically centered around the parietal cortex. The P3 is thought to index brain activities that reflect the updating of the stimulus environment in memory (Donchin, 1981). If the stimulus attributes do not change, as in the case of frequently repeated standard stimuli, then the mental representation of the stimulus does not change. However, when a new stimulus is encountered and processed, attentional mechanisms are engaged that cause the mental representation to be updated, and this is reflected in the P3. The amplitude of this signal is modulated by many different factors and may reflect the number of resources that are allocated to stimulus processing (Kok, 2001; Polich, 2007). To isolate P3 activity, ERPs were averaged over all central, parietal, and parieto‐occipital channels for plotting and statistical analysis.

Second, to investigate neural activity not specific to target processing, EEG data collected during standard’ trials were epoched between 0 and 500 ms, and time–frequency analysis was used to investigate activity in frequency bands associated with different aspects of cognitive processing (delta, theta, alpha, and beta). Trials exceeding ±75 μV across all scalp channels were excluded. Mean trial rejection rates were <4% across all conditions. Event‐related spectral perturbations (ERSPs) were then computed to study event‐related dynamics in the EEG spectrum for frequencies between 1 and 30 Hz (EEGLAB function *newtimef*.*m*). A complex Morlet filter was passed over the data, with the number of wavelet cycles adjusted from one cycle at the lowest frequency to 15 cycles at the highest frequency. The mean of the prestimulus period from −100 to 0 ms was used as a baseline that was subtracted from each poststimulus data sample. The resulting ERSPs spanned from 1 to 30 Hz, with a frequency resolution of 1 Hz and a time resolution of 50 ms. To reduce the number of data points and subsequent statistical comparisons, the data were averaged across epochs and over the theta [4–7 Hz], alpha [9–12 Hz], and beta [18–30 Hz] frequency bands. Frequencies in the lower beta range [14–17.92 Hz] were excluded due to interference from the stimulation frequency. Statistical analyses were restricted to electrode positions at the back of the head [parietal: Pz, P3, P4; parieto‐occipital: POz, PO3, PO4; occipital: Oz, O1, O2].

### 2.9 General statistical approach

Unless otherwise stated, statistical significance for all hypothesis tests was assessed using a nonparametric permutation‐based resampling technique to empirically approximate null distributions for appropriate statistics (Bullock et al., 2017; J. J. Foster et al., 2016; Garcia et al., 2013). This approach has the advantage of being robust to violations of normality. Null distributions were generated according to the type of data being analyzed. Specifically, for the univariate repeated measures analyses, the condition labels were shuffled within participants and 1000 iterations of the appropriate repeated measures ANOVA and pairwise comparisons were computed, which were then used to generate null distributions of *F* values and *t* statistics. Reliable differences were then tested by calculating the probability of obtaining *F* and *t* statistics from each of the null distributions that were greater than the observed *F* and *t* statistics. The standard observed *F* and *t* statistics for each test are reported in the text, along with the critical *p* value (labeled *p*_null_), which represents the probability of observing a value greater than this in the null distribution. To give a more precise sense of the position of the observed statistic in the null distribution, tests are reported as *p*_null_ < 0.05, *p*_null_ < 0.01, or *p*_null_ < 0.001. If *p*_null_ > 0.05 is reported, then the effect was not considered to be statistically significant.

Pairwise comparisons were restricted to the gas challenge manipulations (Hcap, Hpo, Hpox) versus baseline (IsoEu) to reduce the overall number of statistical comparisons. The same technique was applied to regression analyses to investigate the relationship between alpha power and MCAv and PCAv within each gas condition, except that null distributions of *R* ^2^ statistics were generated by shuffling the independent and dependent variable labels. For all time‐course analyses, this technique was applied at each time point and horizontal bars were plotted along the *x*‐axis to indicate significant rest outcomes (*p*_null_ < 0.05). Descriptive statistics reported in the text are presented as mean (*SD*).

## 3 RESULTS

### 3.1 Primary analyses

#### 3.1.1 Behavior

**FIGURE 2:**
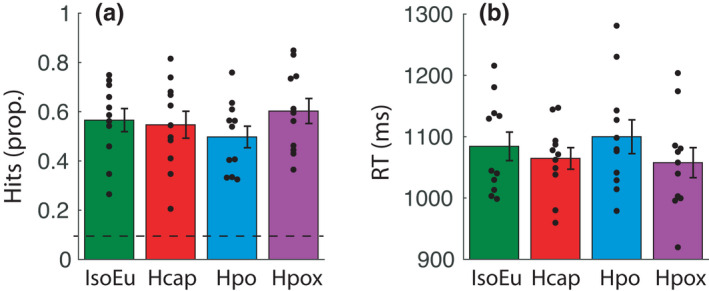
Behavior (a) proportion of “hits” (correctly detected targets). A dashed horizontal line represents chance performance. (b) RTs to correctly detect targets. There was no significant effect of gas condition on hits or RT. Error bars represent ± *SEM*

Gas condition did not modulate target detection performance (*n* = 11). There was no change in hit probability (*p*) [IsoEu: mean = 0.567 (0.157), Hcap: mean = 0.547 (0.182), Hpo: mean = 0.498 (0.145), Hpox: mean = 0.602 (0.168), *F*(3, 30) = 2.68, *p*_null_ > 0.05, *η* ^2^ = 0.21] or Reaction Time (RT; ms) [IsoEu: mean = 1084 (77), Hcap: mean = 1065 (58), Hpo: mean = 1100 (92), Hpox: mean = 1058 (81), *F*(3, 30) = 0.31, *p*_null_ > 0.05, *η* ^2^ = 0.11] (Figure 2a,b, respectively).

#### 3.1.2 End‐tidal traces, global CBFv, and neural responses

**FIGURE 3:**
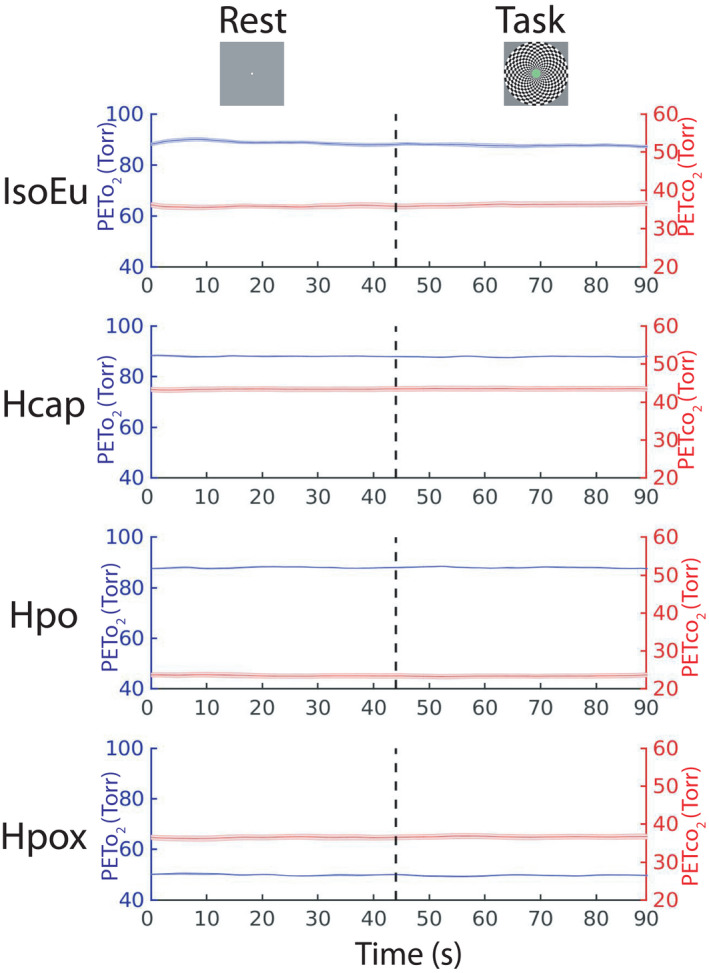
End‐tidal traces. Mean Pet_o_ _2_ and Pet_co_ _2_ traces are plotted, collapsed across all eight 90 s rest (0–45 s)/task (45–90s) cycles for each gas challenge condition, and averaged over all participants. Error bars represent ± *SEM*

**FIGURE 4:**
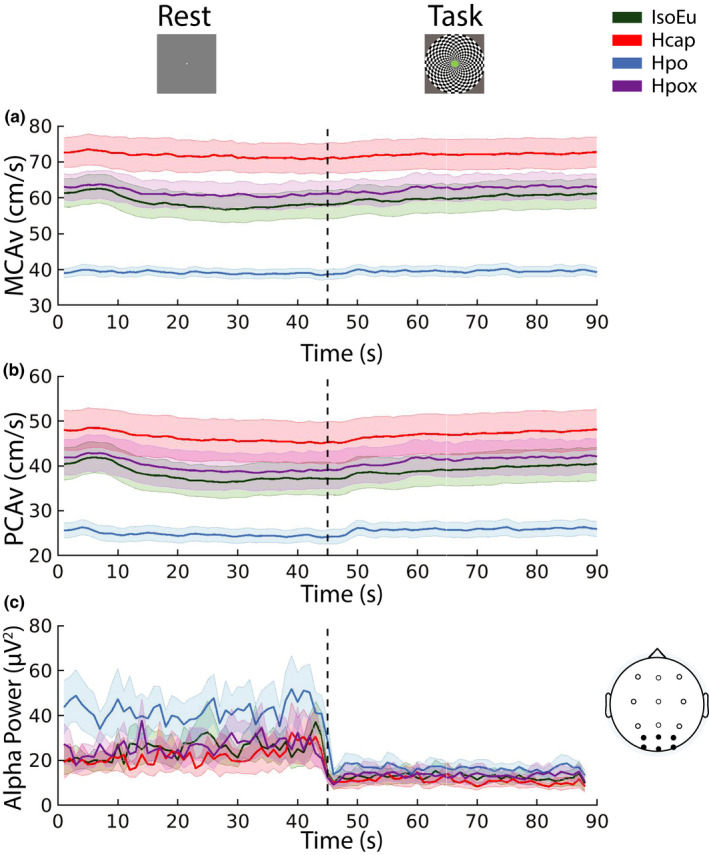
Cerebral blood flow and electroencephalographic activity over rest/task cycle. (a) Middle cerebral artery velocity (MCAv) and (b) posterior cerebral artery velocity (PCAv) were measured over a full 90 s rest/task cycle. (c) Alpha power computed over a full cycle (lines represent mean of PO/O electrode activity). Each line represents data averaged over all participants and trial blocks. Error bars represent ± *SEM*

End‐tidal po_2_ and pco_2_ traces averaged across all eight rest/task cycles are shown in Figure 3. A repeated measures ANOVA (*n* = 12) computed on the averaged final 15 s of the rest and task phases confirmed that end‐tidal po_2_ was modulated by gas challenge [*F*(3, 33) = 7092, *p*_null_ < 0.001, *η* ^2^ = 1] but was not different between the rest phase [IsoEu: mean = 88.09 (1.95), Hcap: mean = 88.09 (0.17), Hpo: mean = 87.90 (0.37), Hpox: mean = 49.87 (0.48)] and the task phase [IsoEu: mean = 87.60 (1.65), Hcap: mean = 87.96 (0.26), Hpo: mean = 87.86 (0.33), Hpox: mean = 49.78 (0.37); *F*(1, 11) = 1.03, *p*_null_ > 0.05, *η* ^2^ = 0.08], and there was no interaction [*F*(3, 33) = 0.53, *p*_null_ > 0.05, *η* ^2^ = 0.05]. A repeated measures ANOVA confirmed that end‐tidal pco_2_ was modulated by gas challenge [*F*(3, 33) = 3209, *p*_null_ < 0.001, *η* ^2^ = 1] and there was also a minimal difference [marginal mean difference = 0.19 (0.05) mmHg] between the rest phase [IsoEu: mean = 35.98 (2.34), Hcap: mean = 43.39 (2.08), Hpo: mean = 23.45 (2.08), Hpox: mean = 36.52 (2.31)] and the task phase [IsoEu: mean = 36.44 (2.25), Hcap: mean = 43.50 (2.17), Hpo: mean = 23.49 (1.97), Hpox: mean = 36.67 (2.24); *F*(1, 11) = 8.23, *p*_null_ < 0.05, *η* ^2^ = 0.43] but no interaction [*F*(3, 33) = 2.38, *p*_null_ > 0.05, *η* ^2^ = 0.18]. MCAv, PCAv, and alpha‐band power averaged across parieto‐occipital/occipital electrodes are plotted over the full 90 s rest/task cycle in Figure 4a–c, respectively. These CBF and EEG data were submitted to statistical analyses in the following sections.

#### 3.1.3 Cerebrovascular and blood pressure responses

##### MCAv

**FIGURE 5:**
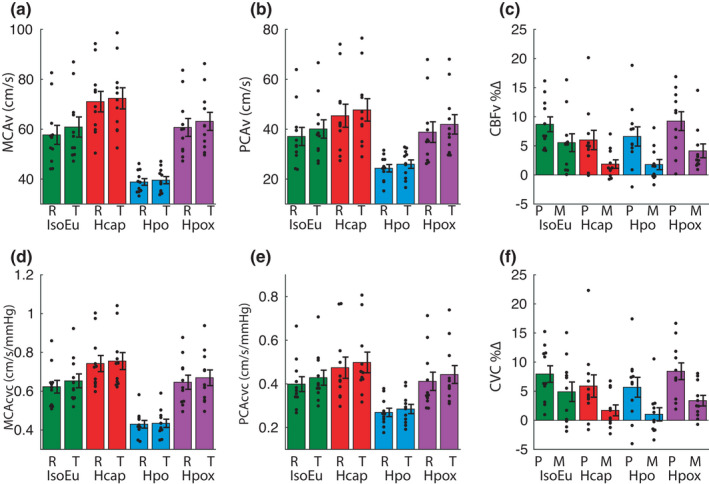
Cerebral blood flow analyses. (a) Middle cerebral artery velocity (MCAv) and (b) posterior cerebral artery velocity (PCAv) averaged over the final 15 s of rest (R) and task (T) phases. (c) Percent change in CBF velocity (CBFv) in PCA (P) and MCA (M) from rest to task phase (normalized to the final 15 s of rest phase). (d) MCA cerebrovascular conductance (MCAcvc) and (e) PCA cerebrovascular conductance (PCAcvc) averaged over the final 15 s of rest (R) and task (T) phases. (f) Percent change in CBFcvc in PCA (P) and MCA (M) from rest to task phase (normalized to the final 15 s of rest phase). Error bars represent ± *SEM*

A repeated measures ANOVA computed for MCAv (*n* = 11) revealed significant main effects of task phase [*F*(1, 10) = 14.29, *p*_null_ < 0.01, *η* ^2^ = 0.59], gas challenge [*F*(3, 30) = 63.52, *p*_null_ < 0.001, *η* ^2^ = 0.86], and an interaction between the task phase and the gas challenge [*F*(3, 30) = 6.05, *p*_null_ < 0.05, *η* ^2^ = 0.38] (Figure 5a). Pairwise comparisons revealed that relative to the IsoEu condition [rest: mean = 57.73 (12.54), task: mean = 60.86 (13.38)], MCAv increased during Hcap [rest: mean = 71.04 (13.63), *t*(10) = −10.79, *p*_null_ < 0.001, *d* = −3.26; task: mean = 72.37 (14.08), *t*(10) = −7.84, *p*_null_ < 0.001, *d* = −2.37], decreased during Hpo [rest: mean = 38.85 (4.36), *t*(10) = 5.91, *p*_null_ < 0.001, *d* = 1.78; task: mean = 39.57 (4.75), *t*(10) = 6.83, p_null_ < 0.001, *d* = 2.06], and increased during Hpox [rest: 60.70 (11.89), *t*(10) = −2.61, *p*_null_ < 0.05, *d* = −0.79; task: mean = 63.13 (12.00), *t*(10) = −2.18, *p*_null_ < 0.05, *d* = −0.66]. Furthermore, MCAv increased during the task phase compared with the rest phase in IsoEu [*t*(10) = −3.78, *p*_null_ < 0.01, *d* = −1.14], Hcap [*t*(10) = −2.49, *p*_null_ < 0.01, *d* = −0.75], and Hpox [*t*(10) = −3.40, *p*_null_ < 0.01, *d* = −1.02] but not Hpo [*t*(10) = −2.10, *p*_null_ > 0.05, *d* = −0.64].

##### PCAv

A repeated measures ANOVA computed for PCAv (*n* = 11) showed significant main effects of task phase [*F*(1, 10) = 53.34, *p*_null_ < 0.001, *η* ^2^ = 0.84] and gas challenge [*F*(3, 30) = 34.89, *p*_null_ < 0.001, *η* ^2^ = 0.78] and an interaction [*F*(3, 30) = 3.50, *p*_null_ < 0.05, *η* ^2^ = 0.26] (Figure 5b). Relative to IsoEu [rest: mean = 37.08 (12.07), task: mean = 40.09 (12.17)], PCAv increased during Hcap [rest: mean = 45.40 (15.34), *t*(10) = −5.81, *p*_null_ < 0.001, *d* = −1.75; task: mean = 47.74 (14.91), *t*(10) = −6.63, *p*_null_ < 0.001, *d* = −2.00], decreased during Hpo [rest: mean = 24.35 (4.99), *t*(10) = 5.26, *p*_null_ < 0.001, *d* = 1.59; task: mean = 26.00 (5.63), *t*(10) = 5.89, *p*_null_ < 0.001, *d* = 1.78], and increased during the task phase but not the rest phase in Hpox [rest: mean = 38.82 (13.62), *t*(10) = −1.60, *p*_null_ > 0.05, *d* = −0.48; task: mean = 41.95 (12.93), *t*(10) = −2.23, *p*_null_ < 0.05, *d* = −0.67]. Furthermore, PCAv increased during the task phase compared with the rest phase in all gas conditions: IsoEu [*t*(10) = −7.79, *p*_null_ < 0.001, *d* = −2.35], Hcap [*t*(10) = −4.55, *p*_null_ < 0.01, *d* = −1.37], Hpo [*t*(10) = −3.87, *p*_null_ < 0.01, *d* = −1.16], and Hpox [*t*(10) = −5.84, *p*_null_ < 0.001, *d* = −1.76].

##### CBF %∆

A 2 [Artery %∆: MCAv, PCAv] × 4 [gas challenge: IsoEu, Hcap, Hpo, Hpox] repeated measures ANVOA (*n* = 11) revealed that PCAv%∆ [IsoEu: mean = 8.70 (4.22), Hcap: mean = 6.00 (5.52), Hpo: mean = 6.61 (5.49), Hpox: mean = 9.24 (5.38)] was consistently greater than MCAv%∆ [IsoEu: mean = 5.54 (5.06), Hcap: mean = 1.87 (2.43), Hpo: mean = 1.78 (2.89), Hpox: mean = 4.14 (3.93), *F*(1, 10) = 11.76, *p*_null_ < 0.001, *η* ^2^ = 0.54] and was modulated by gas challenge CBFv%∆ [*F*(3, 30) = 3.12, *p*_null_ < 0.05, *η* ^2^ = 0.24]. There was no interaction between the two factors [*F*(3, 30) = 0.62, *p*_null_ > 0.05, *η* ^2^ = 0.06] (Figure 5c).

##### MCAcvc

A repeated measures ANOVA for MCAcvc (*n* = 11) revealed main effects of gas challenge [*F*(3, 30) = 67.68, *p*_null_ < 0.001, *η* ^2^ = 0.87], task phase [*F*(1, 10) = 7.68, *p*_null_ < 0.05, *η* ^2^ = 0.43], and an interaction [*F*(3, 30) = 5.01, *p*_null_ < 0.01, *η* ^2^ = 0.34] (Figure 5d). Relative to the IsoEu condition [rest: mean = 0.62 (0.11), task: mean = 0.65 (0.12)], MCAcvc increased during Hcap [rest: mean = 0.74 (0.14), *t*(10) = −6.94, *p*_null_ < 0.001, *d* = −2.09; task: mean = 0.76 (0.15), *t*(10) = −5.59, *p*_null_ < 0.001, *d* = −1.69], decreased during Hpo [rest: mean = 0.43 (0.07), *t*(10) = 7.95, *p*_null_ < 0.001, *d* = 2.40; task: mean = 0.43 (0.07), *t*(10) = 9.16, *p*_null_ < 0.001, *d* = 2.76], and did not change during Hpox [rest: mean = 0.65 (0.12), *t*(10) = −1.30, *p*_null_ > 0.05, *d* = −0.39; task: mean = 0.67 (0.13), *t*(10) = −0.98, *p*_null_ > 0.05, *d* = −0.29]. Furthermore, MCAcvc increased during the task phase compared with the rest phase in IsoEu [*t*(10) = −2.92, *p*_null_ < 0.01, *d* = −0.88] and Hpox [*t*(10) = −3.32, *p*_null_ < 0.01, *d* = −1.00] but not Hcap [*t*(10) = −1.72, *p*_null_ > 0.05, *d* = −0.52] or Hpo [*t*(10) = −0.93, *p*_null_ > 0.05, *d* = −0.28].

##### PCAcvc

A repeated measures ANOVA for PCAcvc (*n* = 11) revealed main effects of gas challenge [*F*(3, 30) = 36.16, *p*_null_ < 0.001, *η* ^2^ = 0.78] and task phase [*F*(1, 10) = 47.56, *p*_null_ < 0.001, *η* ^2^ = 0.83] but no interaction [*F*(3, 30) = 2.39, *p*_null_ > 0.05, *η* ^2^ = 0.19] (Figure 5e). To explore the main effect of gas challenge, the data were averaged across rest and task phases. Relative to the IsoEu challenge [rest: mean = 0.40 (0.11), task: mean = 0.43 (0.11)], PCAcvc increased during Hcap [rest: mean = 0.47 (0.16), task: mean = 0.50 (0.15), *t*(10) = −3.54, *p*_null_ < 0.001, *d* = −1.07], decreased during Hpo [rest: mean = 0.27 (0.06), task: mean = 0.29 (0.07), *t*(10) = 7.24, *p*_null_ < 0.001, *d* = 2.18] and did not change during Hpox [rest: mean = 0.41 (0.14), task: mean = 0.44 (0.14), *t*(10) = −1.09, *p*_null_ > 0.05, *d* = −0.32]. Furthermore, PCAcvc increased during the task phase compared with the rest phase in all gas conditions: IsoEu [*t*(10) = −6.16, *p*_null_ < 0.001, *d* = −1.86], Hcap [*t*(10) = −3.34, *p*_null_ < 0.01, *d* = −1.08], Hpo [*t*(10) = −3.36, *p*_null_ < 0.01, *d* = −1.01], and Hpox [*t*(10) = −6.99, *p*_null_ < 0.001, *d* = −2.11].

##### CBFcvc %∆

A 2 [Artery %∆: MCAcvc, PCAcvc] × 4 [gas challenge: IsoEu, Hcap, Hpo, Hpox] repeated measures ANVOA (*n* = 11) revealed that PCAcvc%∆ [IsoEu: mean = 7.40 (4.70), Hcap: mean = 5.88 (6.38), Hpo: mean = 5.67 (5.67), Hpox: mean = 8.42 (4.78)] was consistently greater than MCAcvc%∆ [IsoEu: mean = 4.98 (5.58), Hcap: mean = 1.70 (3.10), Hpo: mean = 1.02 (3.77), Hpox: mean = 3.37 (3.02), *F*(1, 10) = 11.39, *p*_null_ < 0.01, *η* ^2^ = 0.53], but gas challenge did not modulate CBFcvc%∆ [*F*(3, 30) = 2.12, *p*_null_ > 0.05, *η* ^2^ = 0.12] and there was no interaction between the two factors [*F*(3, 30) = 0.61, *p*_null_ > 0.05, *η* ^2^ = 0.06].

Systolic, diastolic, and mean blood pressure were not different between the rest and task phases in any gas challenge (*p*_null_ > 0.05). As such, changes in cerebrovascular conductance during the task phase generally mimicked those observed for MCAv and PCAv (Figure 5).

#### 3.1.4 Alpha power

##### Alpha (baseline uncorrected)

**FIGURE 6:**
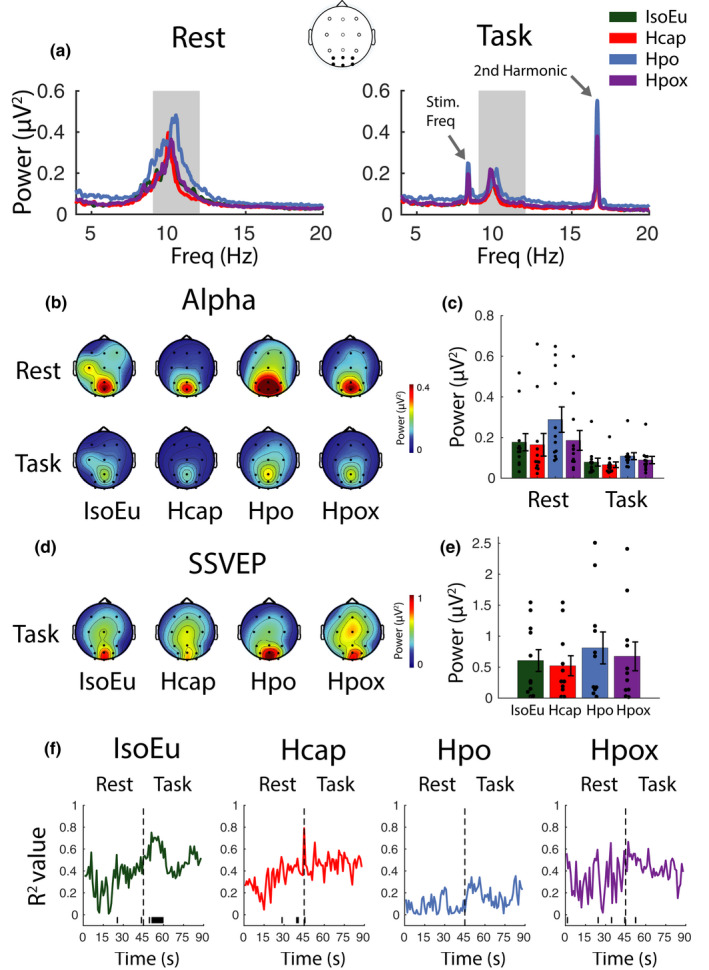
Spectral electroencephalographic and neurovascular coupling analyses. (a) Spectral power during rest and task phases. Data represent the mean of PO/O electrodes. The shaded area represents alpha frequencies used for subsequent calculations. (b) Distribution of alpha across the scalp during rest and task phases. (c) Alpha power (mean of PO/O electrodes). (d) Distribution of steady‐state visual evoked potential (SSVEP) activity [second harmonic; 16.67 Hz] across the scalp during the task phase. (e) SSVEP amplitude (mean of PO/O electrodes). (f) Alpha power regressed against posterior cerebral artery velocity and middle cerebral artery velocity at 1 s intervals across a full rest/task epoch (horizontal line at the base of plot indicates time points, where *p*_null_ < 0.05). Error bars represent ±*SEM*

Spectrograms for the frequency range 4–20 Hz are shown in Figure 6a for both the rest and task phases (*n* = 12). Alpha activation across the scalp is shown in Figure 6b, and mean alpha activity computed across parieto‐occipital/occipital electrodes in Figure 6c. Alpha power was reduced during the task phase compared with the rest phase [*F*(1, 11) = 10.53, *p*_null_ < 0.01, *η* ^2^ = 0.48]. Alpha power was also modulated by gas condition [*F*(3, 33) = 9.02, *p*_null_ < 0.001, *η* ^2^ = 0.45] and there was a significant interaction between the two factors [*F*(3, 33) = 3.47, *p*_null_ < 0.05, *η* ^2^ = 0.24]. Pairwise comparisons determined that alpha power was elevated in Hpo [rest: mean = 0.29 (0.22), task: mean = 0.11 (0.06)] relative to IsoEu [rest: mean = 0.18 (0.15), task: mean = 0.08 (0.07)] both during the rest phase [*t*(11) = −2.56, *p*_null_ < 0.01, *d* = −0.74] and the task phase [*t*(11) = −4.84, *p*_null_ < 0.001, *d* = −1.40]. Alpha power also declined in Hcap [rest: mean = 0.17 (0.19), task: mean = 0.07 (0.05)] relative to IsoEu during the task phase only [*t*(11) = 2.10, *p*_null_ < 0.05, *d* = 0.61]. All other pairwise comparisons were nonsignificant (all *p*_null_ > 0.05).

##### Alpha (baseline corrected)

A repeated measures ANOVA was then computed for the global baseline‐corrected data (*n* = 12) and confirmed the same pattern as for the uncorrected alpha data, such that there was a significant drop in alpha power during the task phase relative to the rest phase [*F*(1, 11) = 13.49, *p*_null_ < 0.001, *η* ^2^ = 0.55], significant modulation by gas condition [*F*(3, 33) = 5.49, *p*_null_ < 0.001, *η* ^2^ = 0.33], and an interaction [*F*(3, 33) = 4.26, *p*_null_ < 0.01, *η* ^2^ = 0.28]. Pairwise comparisons revealed that the interaction was driven by elevated alpha in Hpo [rest: mean = 0.22 (0.21), task: mean = 0.04 (0.07)] relative to IsoEu [rest: mean = 0.14 (0.15), task: mean = 0.03 (0.07)] during the rest phase [*t*(11) = −2.29, *p*_null_ < 0.05, *d* = −0.66] but not the task phase [*t*(11) = −0.72 *p*_null_ > 0.05, *d* = −0.21]. All other pairwise comparisons were nonsignificant (all *p*_null_ > 0.05).

#### 3.1.5 Steady‐state evoked visual responses

##### Steady‐state response (uncorrected)

The effects of gas condition on the steady‐state response at parieto‐occipital/occipital electrodes during the task phase were investigated with a repeated measures ANOVA (*n* = 11) with gas condition [IsoEu, Hcap, Hpo, Hpox] as the within‐participants factor. The test revealed that power at the second harmonic was not significantly modulated by gas condition [IsoEu: mean = 0.61 (0.58), Hcap: mean = 0.52 (0.53), Hpo: mean = 0.81 (0.85), Hpox: mean = 0.68 (0.77), *F*(3, 30) = 1.96, *p*_null_ > 0.05, *η* ^2^ = 0.16] (Figure 6d,e).

##### Steady‐state responses (baseline corrected)

A repeated measures ANOVA (*n* = 11) also confirmed that gas challenge did not modulate SSVEP peak power [IsoEu: mean = 0.58 (0.58), Hcap: mean = 0.50 (0.54), Hpo: mean = 0.76 (0.86), Hpox: mean = 0.65 (0.77), *F*(3, 30) = 1.75, *p*_null_ > 0.05, *η* ^2^ = 0.15]. Note that one participant was removed from these analyses because, due to a technical issue, the checkerboard stimulus used to drive the steady‐state response was presented at a higher frequency compared with other participants, driving a higher frequency response.

#### 3.1.6 Alpha power and CBF relationship

Alpha power was regressed against MCAv and PCAv at each time point over the 90 s rest/task epoch (1 s resolution, *n* = 11) in each of the four gas conditions (Figure 6f). In the IsoEu condition, the *R* ^2^ values increased during the task phase relative to the rest phase, reaching the significance threshold (*p*_null_ < 0.05) for approximately the initial 15 s of the task phase. This suggests that there is a relationship between blood flow in both cerebral arteries and alpha power during engagement in the task. In other gas conditions, the *R* ^2^ values only reached the significance threshold at a few sparsely distributed time points, suggesting that the relationship between alpha and blood flow was disrupted as a function of these arterial blood gas manipulations.

#### 3.1.7 Gas challenge modulates event‐related changes in brain activity

##### P3 ERP component

**FIGURE 7:**
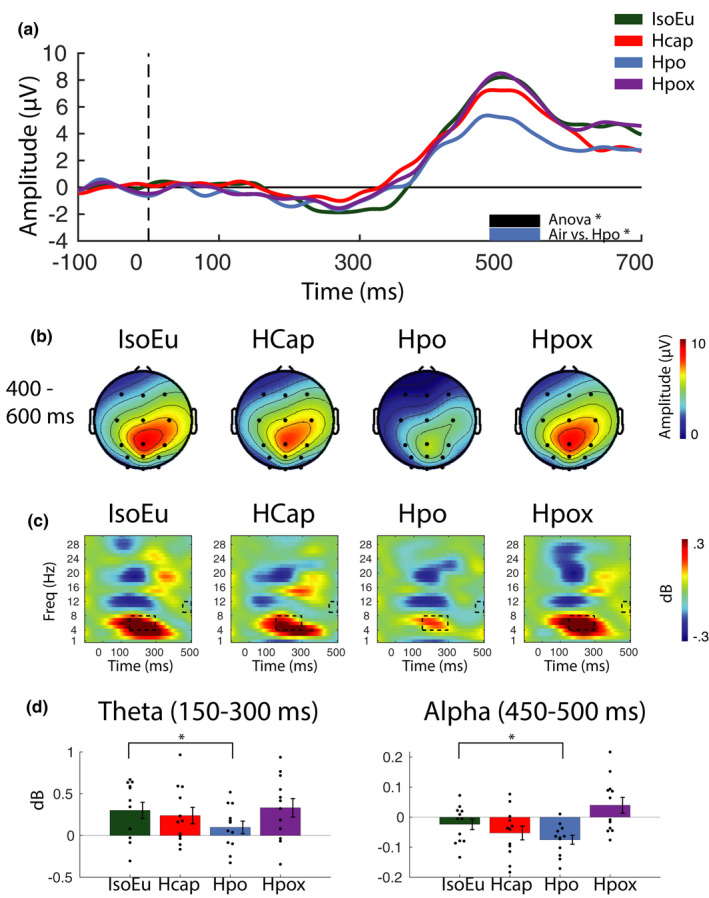
Event‐related electroencephalographic data analyses. (a) Event‐related potentials (ERPs) were computed using mean activation over central, parietal, and parieto‐occipital electrodes for correctly detected target trials. Plots reveal a robust P3 ERP component emerging ~300 ms after the onset of the target period (0 ms). Thick horizontal black and blue bars represent significant ANOVA and pairwise comparisons (*p*_null_ < 0.05). (b) Distribution of P3 ERP activity across the scalp between 400 and 600 ms during correctly detected target trials. (c) Event‐related spectral perturbations (ERSPs) plots show the time course of spectral activation during standard trials in the task phase. Each plot represents activation averaged across parietal and parieto‐occipital and occipital electrodes. Black rectangles denote time–frequency regions that are significantly different according to the ANOVA tests. (d) Bars represent mean activation for regions highlighted in the ERSP plots. Pairwise comparisons indicate that differences in the time–frequency plots are driven by reduced theta and alpha in Hpo relative to IsoEu (*p_null_ < 0.05). Error bars represent ±*SEM*

Examination of the ERPs revealed a robust P3 component centered around electrode Pz (Figure 7a,b) in all gas conditions. Repeated measures ANOVAs (*n* = 12) computed at each time point (averaged over central, parietal, and parieto‐occipital electrodes) confirmed the main effect of gas condition around the P3 peak (~490–560 ms) and pairwise comparisons confirm this effect was driven by reduced amplitude in Hpo compared with the IsoEu condition [mean pairwise comparisons computed across significant time points: *t*(11) = 2.39, *p*_null_ < 0.05, *d* = 0.69]. Gas condition did not modulate P3 peak latency [IsoEu: mean = 520 (16), Hcap: mean = 520 (11), Hpo: mean = 516 (20), Hpox: mean = 520 (21), *F*(3, 33) = 0.01, *p*_null_ > 0.05, *η* ^2^ = 0.001].

##### Time–frequency analysis

Event‐related spectral perturbations were examined to determine whether gas conditions influenced the temporal dynamics of neural activity associated with the processing of standard trial stimuli (Figure 7c,d). Repeated measures ANOVAs (*n* = 12) with the gas condition [IsoEu, Hcap, Hpo, Hpox] as the within‐participant factor were computed, with data averaged across 50 ms time segments, all posterior electrodes (parietal, parieto‐occipital, and occipital sites) and across delta, theta, alpha, and beta frequency bands. The ANOVAs confirmed differences in both theta and alpha bands (*p*_null_ < 0.05) in theta (~150–300 ms poststimulus onset) and alpha (~450–500 ms poststimulus onset). These time–frequency regions are marked with black rectangles in Figure 7c. Follow‐up pairwise comparisons confirmed that the effects in those specific time–frequency regions were driven by reduced theta and alpha in the Hpo condition [mean theta = 0.10 (0.26), mean alpha = −0.08 (0.05)] relative to IsoEu [mean theta = 0.30 (0.34), mean alpha = −0.02 (0.06)], [theta: *t*(11) = 2.44, *p*_null_ < 0.05, *d* = 0.71; alpha: *t*(11) = 1.94, *p*_null_ < 0.05, *d* = 0.56]. Note that target‐hit and target‐miss ERSPs were also computed, but these data were noisy and inconclusive (primarily due to their low trial count), so for the sake of brevity only the standard stimuli were reported here.

### 3.2 Isocapnic‐euoxic hyperventilation control analyses

#### 3.2.1 Behavior

**FIGURE 8:**
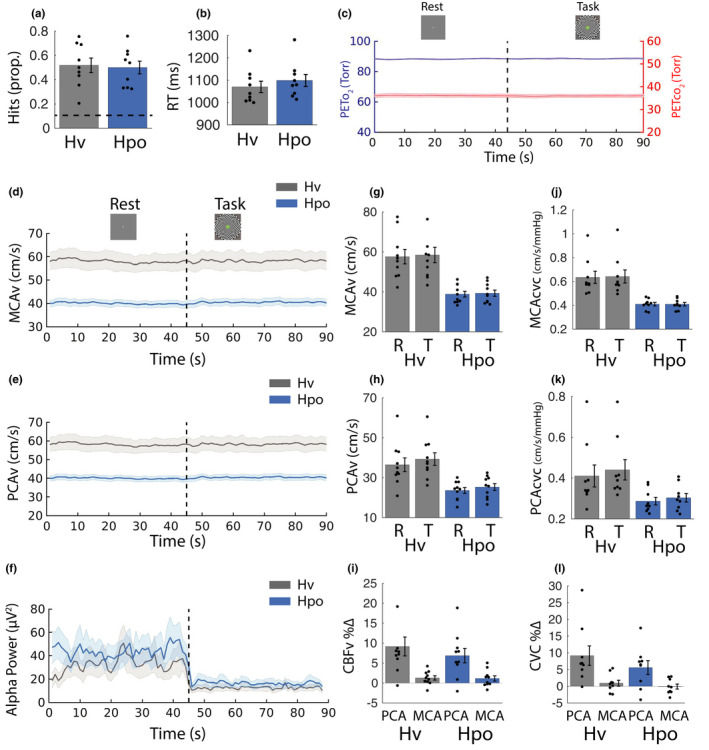
Control analyses. Behavioral performance and physiologic responses were recorded during the isocapnic hyperventilation control condition (Hv) and compared with the hypocapnia condition (Hpo). (a) Proportion of “hits” (correctly detected targets). A dashed horizontal line represents chance performance. (b) RTs to correctly detect targets. (c) Mean Pet_o_ _2_ and Pet_co_ _2_ traces are plotted, collapsed across all eight 90 s rest (0–45 s)/task (45–90 s) cycles for the Hv condition. (d–f) Middle cerebral artery velocity (MCAv), posterior cerebral artery velocity (PCAv), and alpha power (mean of PO/O electrodes), respectively. (g) MCAv and (h) PCAv averaged across the final 15 s of rest and task phases. (i) Percent change in CBFv in PCA and MCA from rest to task phase (normalized to the final 15 s of rest phase). (j) MCA cerebrovascular conductance (MCAcvc) and (k) PCA cerebrovascular conductance (PCAcvc) averaged across the final 15 s of rest and task phases. (l) Percent change in cerebral blood flow cerebrovascular conductance (CBFcvc) in PCA and MCA from rest to task phase (normalized to the final 15 s of rest phase). Error bars represent ±*SEM*

The data from one participant were removed from the behavioral analysis because button press responses were not logged due to a technical issue. Paired *t* tests computed for the remaining participants (*n* = 9) confirmed that hit rate was not modulated by gas challenge [Hv: mean = 0.52 (0.18), Hpo: mean = 0.50 (0.16), *t*(8) = 0.53, *p*_null_ > 0.05, *d* = 0.18] and RT was also not modulated by gas challenge [Hv: mean = 1070 (76), Hpo: mean = 1098 (81), *t*(8) = −0.70, *p*_null_ > 0.05, *d* = −0.23] (Figure 8a,b).

#### 3.2.2 End‐tidal traces, global CBFv, and neural responses

End‐tidal po_2_ and pco_2_ traces for the Hv condition averaged across all eight rest/task cycles and participants are shown in Figure 8c (see Figure 3 for Hpo end‐tidal traces). Pairwise comparisons (*n* = 10) computed for the averaged final 15 s of the Hv rest and task phases confirmed that end‐tidal po_2_ was not different between the rest phase [mean = 88.53 (0.82)] and the task phase [mean = 88.53 (1.21)], *t*(9) = 0.02, *p*_null_ > 0.05. End‐tidal pco_2_ was also not different between the rest phase [mean = 36.04 (2.34)] and the task phase [mean = 36.00 (2.29)], *t*(9) = 0.78, *p*_null_ > 0.05. MCAv and PCAv (Figure 8d,e) and alpha‐band power averaged across parieto‐occipital/occipital electrodes (Figure 8f) are all shown plotted over the full 90 s rest/task cycle. These data were broken down and subject to statistical analyses in the following sections.

#### 3.2.3 Cerebrovascular and blood pressure responses

##### MCAv

A repeated measures ANOVA (*n* = 9) computed for the raw MCAv data revealed higher MCAv during Hv [rest: mean = 55.67 (10.23), task: mean = 56.43 (11.07)] compared with Hpo [rest: mean = 38.01 (4.01), task: mean = 38.43 (4.33), *F*(1, 8) = 32.23, *p*_null_ < 0.001, *η* ^2^ = 0.80] and an increase from rest to task phases across both conditions [*F*(1, 8) = 6.19, *p*_null_ < 0.01, *η* ^2^ = 0.44] but no interaction between phase and condition [*F*(1, 8) = 0.47, *p*_null_ > 0.05, *η* ^2^ = 0.06] (Figure 8g).

##### PCAv

A repeated measures ANOVA (*n* = 9) computed for the raw PCAv data [Hv: rest mean = 35.69 (11.22), task mean = 38.53 (10.19); Hpo: rest mean = 22.91 (4.26), task mean = 24.51 (5.11)] revealed higher PCAv during Hv compared with Hpo [*F*(1, 8) = 21.94, *p*_null_ < 0.001, *η* ^2^ = 0.73] and an increase from rest to task phases [*F*(1, 8) = 32.58, *p*_null_ < 0.001, *η* ^2^ = 0.80] but no interaction [*F*(1, 8) = 1.87, *p*_null_ > 0.05, *η* ^2^ = 0.19] (Figure 8h).

##### CBFv %∆

A repeated measures ANOVA (*n* = 9) computed for the CBF%∆ data revealed PCAv%∆ [Hv: mean = 9.42 (7.89), Hpo: mean = 6.76 (6.04)] was consistently greater than MCAv%∆ [Hv: mean = 1.22 (1.80), Hpo: mean = 1.06 (2.02), *F*(1, 8) = 23.11, *p*_null_ < 0.01, *η* ^2^ = 0.74]. Gas condition did not modulate %∆ in MCAv and PCAv [*F*(1, 8) = 0.43, *p*_null_ > 0.05, *η* ^2^ = 0.05], and there was no interaction between artery and gas condition [*F*(1, 8) = 0.66, *p*_null_ > 0.05, *η* ^2^ = 0.08] (Figure 8i).

##### MCAcvc

A repeated measures ANOVA (*n* = 9) on MCAcvc revealed higher MCAcvc during Hv [rest: mean = 0.63 (0.15), task: mean = 0.64 (0.17)] compared with Hpo [rest: mean = 0.41 (0.04), task: mean = 0.41 (0.04)], supported by a main effect of gas challenge [*F*(1, 8) = 25.77, *p*_null_ < 0.001, *η* ^2^ = 0.76] but no effect of task phase [*F*(1, 8) = 1.15, *p*_null_ > 0.05, *η* ^2^ = 0.13] or interaction [*F*(1, 8) = 0.98, *p*_null_ > 0.05, *η* ^2^ = 0.11] (Figure 8j).

##### PCAcvc

A repeated measures ANOVA (*n* = 9) revealed higher PCAcvc during Hv [rest: mean = 0.41 (0.16), task: mean = 0.44 (0.15)] compared with Hpo [rest: mean = 0.29 (0.06), task: mean = 0.30 (0.06)] supported by a main effect of gas challenge [*F*(1, 8) = 13.00, *p*_null_ < 0.001, *η* ^2^ = 0.62] and task phase [*F*(1, 8) = 25.4, *p*_null_ < 0.001, *η* ^2^ = 0.76] but no interaction [*F*(1, 8) = 2.08, *p*_null_ > 0.05, *η* ^2^ = 0.21] (Figure 8k).

##### CBFcvc %∆

A repeated measures ANOVA (*n* = 9) revealed PCAcvc%∆ [Hv: mean = 9.17 (8.72), Hpo: mean = 5.61 (6.21)] was consistently greater than MCAcvc%∆ [Hv: mean = 0.93 (2.70), Hpo: mean = −0.07 (2.25), *F*(1, 8) = 16.58, *p*_null_ < 0.001, *η* ^2^ = 0.67], but gas challenge did not modulate CBF%∆ [*F*(1, 8) = 1.07, *p*_null_ > 0.05, *η* ^2^ = 0.12], and there was no interaction between the two factors [*F*(1, 8) = 0.49, *p*_null_ > 0.05, *η* ^2^ = 0.06] (Figure 8l).

#### 3.2.4 Alpha power

##### Alpha power (uncorrected)

**FIGURE 9:**
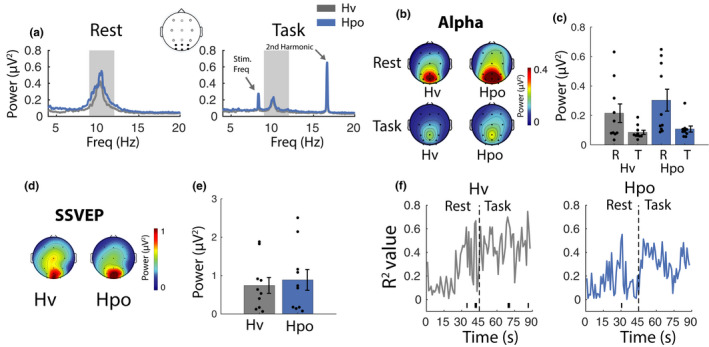
Spectral electroencephalographic and neurovascular coupling control analyses (a) Mean spectral power during rest and task phases. Lines on plot represent the mean of PO/O electrodes. The shaded area represents the alpha frequency band (9–12 Hz). (b) Topographic plots reflect the distribution of alpha activity (9–12 Hz) across the scalp during rest and task phases. (c) Bars represent mean alpha amplitude in each condition (mean of PO/O electrodes). (d) Distribution of steady‐state visual evoked potential (SSVEP) activity (second harmonic; 16.67 Hz) across the scalp during the task phase. (e) SSVEP amplitude (mean of PO/O electrodes). (f) Plots represent alpha power regressed against posterior cerebral artery and middle cerebral artery at 1 s intervals across a full rest/task epoch (horizontal line at the base of plot indicates significant results at *p*_null_ < 0.05). Error bars represent ±*SEM*

Spectrograms for the frequency range 4–20 Hz are shown in Figure 9a for both rest and task phases. A repeated measures ANOVA (*n* = 10) with experiment state [rest, task] and gas condition [Hv, Hpo] as within‐participant factors revealed increased alpha power during Hpo [rest: mean = 0.30 (0.24), task: mean = 0.11 (0.07)] compared with the Hv control [rest: mean = 0.22 (0.20), task: mean = 0.09 (0.05), *F*(1, 9) = 9.17, *p*_null_ < 0.001, *η* ^2^ = 0.50] and reduced alpha power during the task relative to the rest phase [*F*(1, 9) = 8.91, *p*_null_ < 0.001, *η* ^2^ = 0.50] (Figure 9b,c). There was no interaction between the two factors [*F*(1, 9) = 3.16, *p*_null_ > 0.05, *η* ^2^ = 0.26].

##### Alpha power (baseline corrected)

Consistent with the previous analysis, a repeated measures ANOVA (*n* = 10) found increased alpha during Hpo [rest: mean = 0.23 (0.22), task: mean = 0.03 (0.07)] compared with Hv [rest: mean = 0.17 (0.19), task: mean = 0.02 (0.05), *F*(1, 9) = 4.65, *p*_null_ < 0.05, *η* ^2^ = 0.34], reduced alpha power during the task relative to the rest phase [*F*(1, 9) = 12.15, *p*_null_ < 0.01, *η* ^2^ = 0.57], and no interaction [*F*(1, 9) = 3.21, *p*_null_ > 0.05, *η* ^2^ = 0.26].

#### 3.2.5 Steady‐state evoked visual responses

##### Steady‐state (uncorrected)

Power at the stimulation frequency (*n* = 9) was not significantly modulated by gas condition [Hv: mean = 0.73 (0.69), Hpo: mean = 0.91 (0.91), *t*(8) = −1.26, *p*_null_ > 0.05, *d* = −0.42] (Figure 9d,e).

##### Steady state (corrected)

The baseline corrected steady‐state measures (*n* = 9) were also not modulated by gas condition, [Hv: mean = 0.69 (0.70), Hpo: mean = 0.86 (0.91), *t*(8) = −1.19, *p*_null_ > 0.05, *d* = −0.42].

#### 3.2.6 Alpha power and CBF relationship

Alpha power was regressed against MCAv and PCAv at each time point over the 90 s rest/task epoch (1 s resolution, *n* = 9) for both the Hpo and Hv conditions (Figure 9f). Although a few scattered time points did reach the significance threshold, there were no reliable enduring effects during either condition.

#### 3.2.7 Event‐related activity

##### P3 ERP component

**FIGURE 10:**
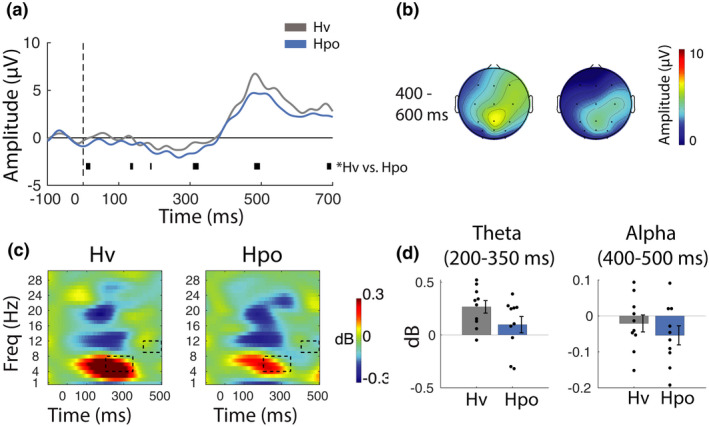
Event‐related electroencephalographic control data analyses. (a) Event‐related potentials (ERPs) were computed using the mean of central, parietal, and parieto‐occipital electrodes for correctly detected target trials. Plots reveal a robust P3 ERP component in both gas conditions. 0 ms represents the onset of the target period. Horizontal bars depict significant statistical tests (*p*_null_ < 0.05). (b) Distribution of P3 ERP activity across the scalp between 400 and 600 ms during correctly detected target trials. (c) Event‐related spectral perturbations (ERSPs) plots show the time course of spectral activation during standard trials in the task phase, averaged across parietal, parieto‐occipital, and occipital electrodes. Black boxes highlight significant differences in theta and alpha activation. (d) Bars represent mean spectral time–frequency activation for regions highlighted in the ERSP plots. Error bars represent ±*SEM*

Examination of the ERPs revealed a robust P3 component centered around electrode Pz (Figure 10a,b) in the two gas conditions (*n* = 10). Paired *t* tests computed at each time point confirmed that the P3 was elevated during Hv compared with Hpo (~500 ms). Gas condition did not modulate P3 peak latency [Hv: mean = 536 (22), Hpo: mean = 532 (20), *t*(9) = 0.17, *p*_null_ > 0.05].

##### Time–frequency analysis

Next, ERSPs were examined to determine whether gas condition influenced the temporal dynamics of neural activity associated with the processing of standard trial stimuli (Figure 10c,d). Paired samples *t* tests (*n*=10) with gas condition (Hv, Hpo) as the within‐participant factor was computed, with data averaged across 50 ms time segments and across the delta, theta, alpha, and beta frequency bands. The tests confirmed differences in both theta and alpha bands (*p*_null_ < 0.05) such that theta (~200–350 ms poststimulus onset) and alpha (~400–500 ms poststimulus onset) were both reduced during Hpo relative to Hv. These time–frequency regions are marked with black rectangles in Figure 10c and plotted in Figure 10d.

## 4 DISCUSSION

The main aims of this study were to (1) assess neurovascular coupling in humans using combined EEG and TCD to measure neural activity and CBF, respectively, at rest and during a cognitive task and (2) investigate how perturbations in arterial blood gasses (i.e., Hcap, Hpo, and Hpox) modulate neurovascular coupling in healthy participants performing an attention task. This novel approach to investigating neurovascular coupling was successful and this experiment yielded new insight into the interaction between resting and task‐related brain activity and CBF and how this is modulated by perturbations in arterial blood flow. Three key sets of findings emerged from the data. First, parieto‐occipital/occipital alpha power was elevated during Hpo relative to IsoEu during both the rest and task phases. Second, the onset of the test phase was accompanied by reduced parieto‐occipital alpha, elevated PCAv in all conditions, and elevated MCAv under all conditions except Hpo. Critically, neurovascular coupling between alpha and PCAv/MCAv was observed immediately after task onset but only during the isocapnic‐euoxia condition and not during Hcap, Hpo, or Hpox conditions. Third, brain activity directly related to stimulus processing in the attention task was attenuated during Hpo relative to IsoEu. These findings are discussed with reference to the extant literature documenting the effects of perturbations of arterial blood gases on resting‐state cerebral activity as well as work examining the dynamics of alpha and theta band activation during attention.

### 4.1 Alpha oscillations

Increased alpha power reflects synchronization of the alpha rhythm, which is thought to reflect active inhibition of cognitive processing and reduced attentional allocation (e.g., Jensen & Mazaheri, 2010; Klimesch et al., 2007; Mathewson et al., 2011; Thut et al., 2006; but see Foster & Awh, 2019). The present data, therefore, suggest increased inhibition and reduced attentional allocation during Hpo relative to IsoEu, although it is important to note these effects were not accompanied by any significant decline in behavioral performance in our sustained attention task. In the Hpo challenge, Pet_co_ _2_ was decreased by verbally coaching participants on the rate and depth of breathing in order to maintain Pet_co_ _2_ ~12 mm Hg below air‐breathing values. The modulations observed in alpha are generally consistent with previous investigations that also used acute hyperventilation to induce hypocapnia in participants and showed increased theta and alpha amplitude across the entire lower EEG spectrum and over the entire scalp compared with normal breathing (Kennealy et al., 1986; Zwiener et al., 1998). Importantly, control analyses in the present study show elevated alpha during Hpo compared with Hv, confirming that the effects in alpha are due to reduced arterial pco_2_ and not the act of hyperventilation per se.

Although the most robust effects were observed during Hpo, alpha was also reduced during Hcap relative to IsoEu; a finding that is consistent with previous EEG and magnetoencephalography recordings from participants at rest (Hall et al., 2011; Wang et al., 2014; Xu et al., 2011). The cause of this desynchronization is unclear, although one hypothesis proposes that the pH decrease caused by Hcap can reduce neuronal excitability, which could reduce spiking rates and thus impact upon local field potentials (Hall et al., 2011; Zappe et al., 2008). The converse of this effect may also account for alpha effects during Hpo, such that increased pH during Hpo may increase neuronal excitability and spiking rates, thus enhancing local field potentials (Ruusuvuori & Kaila, 2014, pp. 271–290).

### 4.2 CBF and neurovascular coupling

The overall pattern of CO_2_‐specific gas condition effects on CBF is consistent with previous studies showing cerebral vasodilation and increased CBF during Hcap (Ances et al., 2001; Flück et al., 2014; Maggio et al., 2013, 2014; Poulin et al., 1996; Schmitz et al., 1996) and vasoconstriction and decreased CBF during Hpo (Ito et al., 2000, 2003; Raper et al., 1971; Shapiro et al., 1970) compared with air‐breathing and isocapnic‐euoxia conditions. Consistent with previous data, MCAv increased during Hpox relative to IsoEu (Beaudin et al., 2011; G. E. Foster et al., 2007; Poulin et al., 1996) during both rest and task phases; however, PCAv only increased during Hpox in the task phase and not the rest phase. The lack of Hpox effects on PCAv during the rest phase may be attributed to PCA being not as responsive to Hpox because it perfuses a smaller cerebral area (Kellawan et al., 2017).

The onset of visual stimulation in each task phase prompted a rapid and consistent increase in PCAv across all gas challenge conditions, suggesting that the visually evoked relative flow velocity increase (i.e., % change in blood flow velocity in relation to preceding rest phase) is maintained despite the large changes in baseline CBF induced by the different gas conditions. This finding is novel for hypoxia and hypocapnia in relation to PCAv as, to our knowledge, no previous work has investigated neurovascular coupling in response to a visual challenge under these conditions. However, the enduring increase in PCAv during hypoxia is similar to previous observations for MCAv increases during neural activation, where MCAv increased by ~7%–8% during the performance of a Stroop task within both normoxia and hypoxia (Lefferts et al., 2016). The enduring PCAv response during hypercapnia is also consistent with an fMRI study that demonstrated the absolute ∆CBF response to visual stimulation is constant across normocapnia and Hcap conditions (Whittaker et al., 2016). Therefore, as neuronal activation induced a similar proportional increase in CBF within all gas conditions it appears that in younger, healthy individuals neurovascular coupling is not impaired by altered levels of vasodilation and vasoconstriction resulting from changes in arterial blood gases. Whether this relationship is maintained into older age, under longer duration exposure to blood gas alterations (e.g., travel to altitude), or in patients with chronic medical conditions associated with alterations in arterial blood gases (e.g., OSA, COPD) still needs to be investigated.

Visually evoked flow velocity increase was also observed for the MCA during the IsoEu, Hcap, and Hpox conditions, but similar to our previous study during normoxia (Flück et al., 2014), the increase was several orders of magnitudes smaller compared with the PCA responses. This is not unexpected as monitoring blood flow through the MCA during a visual challenge typically acts as a negative control as the MCA does not perfuse the visual cortex (Aaslid, 1987; Flück et al., 2014). The fact that MCAv did not increase as a function of visual stimulation during the Hpo condition suggests that perturbations in arterial CO_2_ may disrupt visually evoked velocity increase in MCA.

In addition to the blood flow effects, the onset of visual stimulation prompted a rapid reduction in parieto‐occipital/occipital alpha power across all gas conditions. Neurovascular coupling between alpha and both cerebral arteries was observed almost immediately after task onset during isocapnic‐euoxia, and this relationship was sustained for the initial ~15 s of the task phase (Figure 6f). It is unclear why the relationship is not sustained for the entire duration of the task phase, but this may indicate that strong coupling is only present while CBF is being adjusted to support the task demands. In contrast, coupling was not observed during any of the experimental conditions, which may reflect the decoupling of neural and cerebrovascular responses as a function of the arterial blood gas manipulations.

Overall, these data confirm that activation of the visual cortex is associated with reduced parieto‐occipital/occipital alpha power and increased PCA blood flow. While vasodilation/vasoconstriction caused by our gas challenges does not appear to influence the visually evoked flow velocity increase in the PCA, there appear to be CO_2_‐specific effects on the MCA response.

### 4.3 Event‐related neural activity

Event‐locked perturbations in brain activity were analyzed to determine whether specific stages of cognitive processing were impacted by alterations in blood gasses. The P3 ERP component induced by correctly identified target trials was reduced in amplitude during Hpo relative to IsoEu. P3 amplitude can be modulated by many different factors, but converging evidence from a number of P3 studies suggests that P3 amplitude decreases when a task is made more difficult by making a stimulus harder to discriminate (Kok, 2001). This has led to the suggestion that P3 amplitude represents the amount of information transmitted during stimulus presentation, which is inversely related to the participant's uncertainty at having correctly perceived the event (e.g., Johnson, 1988; Kok, 1997, 2001). In the present study, the physical characteristics of the stimulus are not directly manipulated, but it is possible that during Hpo, participants find stimulus discrimination more challenging, which results in reduced P3 amplitude. Hypocapnia is associated with reduced CBF and it is plausible that this could impact cognitive processing. Previous work investigating hypocapnia effects on the P3 has demonstrated mixed results. One study found reduced P3 amplitude under acute heat stress, where hypocapnia develops secondary to hyperventilation (Shibasaki et al., 2016); however, investigations where hypocapnia was induced by mechanical ventilation (Bloch‐Salisbury et al., 2000) report no change in amplitude. Although, in the present study, there is a robust reduction in P3 amplitude in Hpo relative to IsoEu, it is important to note that the effect is much weaker in the control study comparing Hpo with Hv. Furthermore, while perturbations in blood gasses have been associated with impaired cognitive functioning across multiple cognitive domains (e.g., McMorris et al., 2017; Schou et al., 2012), there are no significant behavioral effects in our sustained attention task, although we note that mean performance does tend to be lower in Hpo in both main and control analyses.

Previous work has demonstrated slowing of the P3 latency as a function of either acute or chronic hypoxic exposure (Fowler & Kelso, 1992; Fowler & Prlic, 1995; Hayashi et al., 2005; Singh et al., 2003; Thakur et al., 2011) but no similar effects were observed here. One possible explanation for this discrepancy is that previous studies used a standard “oddball” task to elicit a P3, where participants are required to respond to oddball stimuli that differ from standard stimuli by a salient feature such as visual stimulus intensity or tone frequency and this may result in more homogeneous P3 latencies across trials. In the version of the task used in the current study, participants discriminated oddball targets from standards based on duration, which is not discrete and may result in more latency variability on individual trials, meaning the latency measures are less sensitive to any effects of the experimental manipulation.

Time–frequency analyses of the standard (i.e., nontarget) trials revealed suppression of theta and alpha burst activity approximately 150–300 ms and 450–500 ms poststimulus, respectively, during Hpo compared with IsoEu. Enhanced bursts of theta in parieto‐occipital cortex are associated with suppression of task‐irrelevant information (Green & McDonald, 2008) and increased task‐switching demands (Gladwin & de Jong, 2005), hence the theta suppression observed during Hpo may reflect interference with these cognitive processes. Increased alpha power is associated with active inhibition of cognitive processing and reduced attentional allocation (e.g., Klimesch et al., 2007; Mathewson et al., 2009; O’Connell et al., 2009), therefore, suppression of alpha late in the trial (450–500 ms) implies the release of inhibition in preparation for the next stimulus in the sequence. Baseline alpha power is elevated during Hpo, so this might explain why alpha suppression is much greater during Hpo compared with IsoEu, as the brain has to overcompensate with greater release from inhibition in an attempt to ensure successful stimulus processing. Both theta and alpha effects are also present in the control study, confirming that these effects are not just an artifact of hyperventilation.

Together, these effects show task‐specific modulation of neural activity across multiple frequency bands as a function of hypocapnia and suggest that the effects of hypocapnia on the brain may not be uniform.

### 4.4 Methodologic considerations, limitations, and potential applications

To our knowledge, this study is the first to combine EEG and TCD in the context of assessing neurovascular coupling. The advantage of TCD is that it has excellent temporal resolution and has been used for >30 years for noninvasive assessment of neurovascular coupling. Furthermore, TCD has also been used to assess CBF responses to cognitive, verbal, and motor tasks (Willie et al., 2011; Wolf, 2015). Combined with the excellent temporal resolution of EEG, these techniques provide a promising method for studying the temporal dynamics of neurovascular coupling. One caveat of this approach is that both EEG and TCD suffer from poor spatial resolution. The scalp is a volume conductor and this makes it difficult to determine the specific neural source of EEG activity, and with TCD, it is only possible to infer that blood flow is modulated in the brain region perfused by the insonated artery. An alternative approach combining arterial spin labeling (an MRI technique that enables the quantification of CBF through the brain) with EEG could potentially provide more precise regional blood flow information, however, collecting these data would be considerably more technically complicated and expensive.

Nevertheless, these data suggest that simultaneous EEG and TCD can be successfully combined with assessing neurovascular coupling in healthy adults. Given the novelty and exploratory nature of this approach, healthy normotensive individuals were recruited to better assess the relationship between these two modalities during acute exposure to alterations in arterial blood gases, prior to applying this technique to patient populations. It must be acknowledged that the current study is limited in scope and that this methodology does require further validation with large, diverse samples to comprehensively assess neurovascular coupling. Furthermore, it is also important to acknowledge that a poikilocapnic hypoxia gas challenge condition was not included as part of the study. This was because the objective of the present study was to assess the independent changes of arterial oxygen and CO_2_ on the responses. Furthermore, this was already a long experimental session for each participant (5+ h) and it did not seem reasonable to extend it with additional conditions.

A natural progression of study would study the effects of prolonged alterations of arterial blood gasses, such as extended exposure to hypoxia (G. E. Foster et al., 2015; Villien et al., 2013) or OSA (Busch et al., 2016; Tekgol Uzuner & Uzuner, 2016), as many adaptive/compensatory mechanisms can occur that affect the normal physiologic responses and even the cerebral structure. For this study, a simple attention task was chosen because attention underpins many higher‐order cognitive functions and this was considered to be an important first target for investigation. Future work might assess neurovascular coupling in other cognitive domains by using tasks designed to tap higher cognitive functions such as the Stroop task for measuring inhibitory control or the *n*‐back task for assessing working memory performance (Jaeggi et al., 2010; McLeod, 1991).

## 5 CONCLUSION

This study investigated the extent to which perturbations in arterial blood gases influence the cognitive function of attention and the underlying neural activity in healthy volunteers. Relative to baseline (isocapnic‐euoxia), effects of Hcap, Hpo, and Hpox were observed on both spontaneous and task‐related neural activity as measured by EEG. First, there was evidence for modulation of alpha‐band activity as a function of both Hpo and Hcap. Second, there was evidence for CO_2_‐specific effects on the relationship between neuro‐electrical activity and the cerebrovascular response. Third, there was task‐specific modulation of theta activity in the parieto‐occipital cortex during Hpo, suggesting that Hpo effects on the brain may not be global and uniform. Future work can use the noninvasive multimodal approach outlined in this study to investigate how perturbations in arterial blood gases influence a range of different cognitive functions and the underlying neural and cerebrovascular activity.

## CONFLICT OF INTEREST

The authors declare no competing financial interests.

## AUTHOR CONTRIBUTIONS

All authors were involved in the conception and design of the work. AEB informed, instructed, and gained the volunteers’ consent to participate. AEB anonymized the data and analyzed the physiologic data. TB and BG analyzed the anonymized EEG data, performed the regression analysis, and conducted the statistical tests. All authors wrote and revised the manuscript. All authors approved the final version of the manuscript. All authors agree to be accountable for all aspects of the work in ensuring that questions related to the accuracy or integrity of any part of the work are appropriately investigated and resolved. All persons designated as authors qualify for authorship, and all those who qualify for authorship are listed.
